# Isolation of DiNP-Degrading Microbes from the Mouse Colon and the Influence DiNP Exposure Has on the Microbiota, Intestinal Integrity, and Immune Status of the Colon

**DOI:** 10.3390/toxics10020075

**Published:** 2022-02-06

**Authors:** Karen K. Chiu, Shah Tauseef Bashir, Ahmed M. Abdel-Hamid, Lindsay V. Clark, Mary J. Laws, Isaac Cann, Romana A. Nowak, Jodi A. Flaws

**Affiliations:** 1 Division of Nutritional Sciences, College of Agricultural, Consumer and Environmental Sciences, University of Illinois, Urbana, IL 61801, USA; kkchiu2@illinois.edu; 2 Department of Comparative Biosciences, College of Veterinary Medicine, University of Illinois, Urbana, IL 61802, USA; marylaws@illinois.edu; 3 Department of Molecular and Integrative Physiology, College of Liberal Arts & Sciences, University of Illinois, Urbana, IL 61801, USA; tbshah3@illinois.edu (S.T.B.); icann@illinois.edu (I.C.); 4 Department of Animal Sciences, College of Agricultural, Consumer and Environmental Sciences, University of Illinois, Urbana, IL 61801, USA; ranowak@illinois.edu; 5 Carl R. Woese Institute for Genomic Biology, University of Illinois, Urbana, IL 61801, USA; ahetta@illinois.edu; 6 High Performance Computing in Biology, Roy J. Carver Biotechnology Center, University of Illinois, Urbana, IL 61801, USA; lvclark@illinois.edu

**Keywords:** di-isononyl phthalate (DiNP), gut microbiota, immunology, tight junctions

## Abstract

Di-isononyl phthalate (DiNP) is a plasticizer used to impart flexibility or stability in a variety of products including polyvinyl chloride, cable coatings, artificial leather, and footwear. Previous studies have examined the impact of DiNP on gut integrity and the colonic immune microenvironment, but this study further expands the research by examining whether DiNP exposure alters the colonic microbiota and various immune markers. Previous studies have also revealed that environmental microbes degrade various phthalates, but no studies have examined whether anaerobic gut bacteria can degrade DiNP. Thus, this study tested the hypothesis that DiNP exposure alters the gut microbiota and immune-related factors, and that anaerobic bacteria in the gut can utilize DiNP as the sole carbon source. To test this hypothesis, adult female mice were orally dosed with corn oil or various doses of DiNP for 10–14 consecutive days. After the treatment period, mice were euthanized during diestrus. Colonic contents were collected for full-length 16S rRNA gene sequencing to identify the bacteria in the colon contents. Sanger sequencing of the 16S rRNA gene was used to identify bacteria that were able to grow in *Bacteroides* minimal media with DiNP as the sole carbon source. Colon tissues were collected for immunohistochemistry of immune(-related) factors. An environmentally relevant dose of DiNP (200 µg/kg) significantly increased a *Lachnoclostridium* taxon and decreased *Blautia* compared to the control. Collectively, minimal changes in the colonic microbiota were observed as indicated by non-significant beta-diversities between DiNP treatments and control. Furthermore, three strains of anaerobic bacteria derived from the colon were identified to use DiNP as the sole carbon source. Interestingly, DiNP exposure did not alter protein levels of interleukin-6, tumor necrosis factor alpha, claudin-1, and mucin-1 compared to the control. Collectively, these findings show that DiNP exposure alters the gut microbiota and that the gut contains DiNP-degrading microbes.

## 1. Introduction

The gut microbiome is defined as a collection of microorganisms that reside within the gastrointestinal tract along with its genetic material [1]. It is a complex ecosystem and plays essential roles in intestinal homeostasis, function, protection, and immunology. As abundant are the roles of the gut microbiome, so are the factors that influence the gut microbiome. Diet, the birthing process, age, sex, psychological stress/anxiety, medication/drug use, and physical activity are all factors that influence the gut microbiome. Interestingly, environmental factors also impact the gut microbiome [2]. Exposure to environmental chemicals such as heavy metals, pesticides, persistent organic pollutants, and plasticizers all perturb the gut microbiome [3]. In particular, phthalate exposures impact the gut microbiome in newborn infants, rats, mice, and fish during puberty, birth, and pregnancy [4,5,6,7,8,9].

Phthalates are a large class of chemicals that function as stabilizers and plasticizers and are further divided into low- and high-molecular weight phthalates. The size of the phthalate can determine its function and applications. For example, low-molecular weight phthalates with three to six carbons (e.g., DMP, DEP, diisobutyl phthalate (DIBP), and DBP) are commonly used as stabilizers in personal care products (e.g., perfume, lotions, body washes, shampoo), whereas high-molecular weight phthalates with more than six carbons (e.g., DEHP and di-isononyl phthalate (DiNP)) are commonly used as plasticizers to ensure polymers are durable and malleable [10]. In this regard, high-molecular weight phthalates are commonly used in polyvinyl chloride, construction, building materials, cable wires, medical equipment, children’s toys, and vinyl clothing. The current study focuses on a high-molecular weight phthalate called di-isononyl phthalate (DiNP). Studies have shown that DiNP is an endocrine-disrupting chemical that disrupts sex steroid hormones and reproductive organs [11]. Studies have also shown that endocrine-disrupting phthalates alter the gut microbiome, resulting in unintended consequences, especially since the gut microbiome is the largest virtual endocrine organ [12].

Although it is known that phthalates, such as DBP, DEHP, DEP, DMP, and MEHP alter the gut microbiome, limited information was available about the extent that DiNP impacts the gut microbiome, specifically the colonic microbiome. The colon was examined in this study because it harbors the most bacteria, and it has a special function of keeping bacteria at bay without expelling bacteria out of the gut by keeping microbes trapped in the mucus layer. Furthermore, previous studies have shown that phthalates can be degraded by microbes found in the marine environment, sediment, soil, wastewater, and activated sludge [13,14,15]. However, it was still unknown whether gut microbes are capable of degrading or using phthalates, specifically DiNP, as the sole carbon source. Therefore, the purpose of this study was to determine the extent to which DiNP exposure impacts the colonic microbiome, identify bacteria residing in the mouse colon that use DiNP as a carbon source, and to determine whether DiNP exposure impacts immune factors in the gut.

## 2. Materials & Methods

### 2.1. Chemicals

DiNP (Sigma-Aldrich, St. Louis, MO, USA) was dissolved in corn oil (MP Biomedicals, Solon, OH, USA). The corn oil was used as a solvent for DiNP, and it was also used as a vehicle control (0 µg/kg DiNP). Environmentally relevant doses of DiNP were selected for this study, which included 20 and 200 µg/kg of DiNP. In detail, 20 µg/kg of DiNP was selected to represent exposure in occupational workers [16,17,18], whereas 200 µg/kg of DiNP was selected to represent exposure in infants and children 0–18 months [19]. In addition to these environmentally relevant doses, we also included 2, 20, and 200 mg/kg doses of DiNP for immunohistochemistry experiments. These doses were selected to determine the dose-response curve for DiNP and to compare with other studies using doses of DiNP within this range [11,20,21,22].

### 2.2. Experimental Animals and Design

All animal care and use procedures were approved on 24 June 2019 by the University of Illinois Institutional Animal Care and Use Committee prior to experimentation (Protocol No.: 20,034 and 19,110) and performed in AAALAC (Association for Assessment and Accreditation of Laboratory Animal Care)-approved animal facilities.

In the first animal experiment for microbial determination, female CD-1 mice (6 weeks of age; Charles River) were purchased and single-housed in standard cages in an environmentally controlled room (12-h light: 12-h dark cycle, 50 ± 20% humidity, and 21.1 ± 2.2 °C). Upon arrival, mice were fed water and a standard 2918 Tekland diet *ad libitum*. The mice were also acclimated to the facilities for 14 days. During the acclimation period, bedding from each cage was consolidated and mixed before redispersing the mixed bedding to each cage. This process of mixing the bedding was repeated every 7 days; therefore, the process was performed two times over the course of the acclimation period. After acclimation, mice were randomly assigned to 3 groups (*n* = 5/group): corn oil (control), 20 µg/kg DiNP, or 200 µg/kg DiNP. Animals were orally dosed by gently pipetting corn oil vehicle, 20 µg/kg of DiNP, or 200 µg/kg of DiNP once a day for 10 days. After 10 days of DiNP exposure, mice were euthanized by CO_2_ asphyxiation followed by cervical dislocation during diestrus, and colon contents were collected. Diluted phosphate-buffered saline (PBS) was used for vaginal cytology examination to determine their estrous cycle. If mice were not in diestrus on day 10, they were continually dosed until they reached diestrus. However, the mice that were not in diestrus at day 10 were not incorporated in the analysis of 16S rRNA sequencing data.

A separate animal experiment was carried out exactly like previously published studies to examine immune and immune-related factors in the colon tissues and expand on these previous studies [3,23]. Briefly, female CD-1 mice (approximately 2 months of age) were group-housed and orally dosed with corn oil (control), 20 µg/kg, 200 µg/kg, 2 mg/kg, 20 mg/kg, or 200 mg/kg of DiNP for 10–14 consecutive days. At the end of the dosing period, mice were euthanized in diestrus, and colon tissues were collected for immunohistochemistry (IHC). Mice that were not in diestrus after 10 days of dosing were continually dosed for a maximum of 14 days until they reached diestrus.

### 2.3. Tissue Collection

Colon tissues were trimmed to remove mesenteric tissues, flushed of colonic contents, and fixed in 10% formalin (Macron Fine Chemicals, Center Valley, PA, USA) overnight. The following day, the formalin solution (Sigma-Aldrich, St. Louis, MO, USA) was replaced with 70% ethanol at 4 °C until further processing. Colon tissues were further processed into formalin-fixed paraffin-embedded (FFPE) blocks and sliced into 7 µm thick sections using a microtome (Microm HM310). The sections were then mounted onto Surgipath A-Tra Microscope glass slides (Leica, Lincolnshire, IL, USA).

### 2.4. Immunohistology and Analysis

FFPE slides were subjected to immunohistochemistry (IHC) as described by Chiu et al. [23] Briefly, sides were deparaffinized using xylene and ethanol. Sodium citrate buffer (pH 6.0) was used for antigen retrieval, and 0.3% hydrogen peroxide was used to block endogenous peroxidases. Tissues then were blocked with 5% bovine albumin serum and 10% normal goat serum (NGS) for 1 h. Primary antibody was incubated overnight at 4 °C in a humidity chamber. The primary antibodies used in the current study were anti-CD3 (ab5690, 1:500), anti-CLDN1 (Invitrogen 71-7800, 1:2000), anti-IL-6 (ab208113, 1:500), anti-MUC1 (Invitrogen PA5-95487, 1:50), and anti-TNF alpha (Invitrogen AMC3012, 1:1000). The following day, slides were washed, incubated with the appropriate secondary antibody, and developed with 3,3′-diaminobenzidine (Vector Laboratories, SK4100). Hematoxylin was used to counterstain, and hematoxylin intensity was equal across all slides and samples. Slides were scanned and imaged using NDP.scan 3.2.15 and Hamamatsu NanoZoomer 2.0 HT (Model No. C9600-12). Quantification was carried out on FIJI. The person performing the analysis was blinded to the treatment group.

### 2.5. Anaerobic Diluent and Colon Content Collection

Anaerobic diluent was used as a medium to collect the gut microorganisms under strict anaerobic conditions. Anaerobic diluent was created according to McSweeny et al. with modifications [24]. Briefly, all the components of the anaerobic diluent (Table 1) except cysteine HCl were mixed together, heated until boiling, and saturated with CO_2_ using gassing probes. After gassing, anaerobic diluent was transferred into the anaerobic chamber, and cystine HCl (1 g/L) was added. Anaerobic diluent was dispensed into 9.0 mL aliquots into anaerobic Balch tubes. The Balch tubes were capped with rubber stoppers, then sealed with aluminum seals, and finally, autoclaved at 121 °C for 15 min.

Colonic contents were collected within 15 min of euthanization and obtained by flushing sterile anaerobic diluent through the colon. The colonic contents were resuspended in anaerobic diluent and injected into Balch tubes containing 9 mL of anaerobic diluent.

### 2.6. Isolation and Characterization of DiNP-Degrading Bacteria

Within 2 h of colon content collection, 1 mL of anaerobic diluent containing the colonic contents was injected into another anaerobic Balch tube containing sterile *Bacteroides* minimal media with DiNP. Cultures were incubated at 37–38 °C. Mass spectrometry analysis was conducted by the Metabolomics Lab of the Roy J. Carver Biotechnology Center at the University of Illinois Urbana-Champaign to measure levels of DiNP in the inoculated *Bacteroides* minimal media. Mass spectrometry procedures were carried out according to previously defined methods [25,26] and used for confirming the degradation of DiNP by microbes (data not shown).

### 2.7. Bacteroides Defined Minimal Media

The *Bacteroides* defined minimal media were created following a protocol originally published by Varel and Byrant [27], with some modifications (Table 2). Specifically, the following solutions or chemicals, expressed as milliliters or grams per liter (mg/L or g/L), were used to make the defined minimal media: mineral 3B solution (50 mL), cysteine hydrochloride (1 g), hemin solution (10 mL), 0.01% vitamin B12 (1 mL), 10% DiNP solution (10 mL), iron (II) solute (FeSO_4_) solution (1.5 mL), 7% NaHCO_3_ (14.4 mL), 0.1% resazurin (1 mL) and water. The ingredients used to make the mineral 3B solution consisted of the following (expressed in g/L): KH_2_PO_4_ (18 g), NaCl (18 g), MgCl_2_•6H_2_O (0.4 g), CaCl_2_•2H_2_O (0.52 g), CoCl_2_•6H_2_O (0.02 g), MnCl_2_•4H_2_O (0.2 g), NH_4_Cl (10 g), and Na_2_SO_4_ (5 g). Hemin solution was prepared by dissolving 100 mg of hemin in 2 mL of 1 M NaOH and bringing up the volume to 200 mL with dH_2_O. Unlike the in vivo portion of the current study in which DiNP was dissolved in corn oil, DiNP was dissolved in DMSO for the bacterial cultures as corn oil could be a nutrient source for microbes. FeSO_4_ solution was created by dissolving 0.278 g of FeSO_4_•7H_2_O in 100 mL dH_2_O. To help with the dissolving process, two drops of concentrated HCl were added to the FeSO_4_ solution.

Mineral 3B solution, hemin solution, FeSO_4_ solution, resazurin, and water were mixed together and then boiled under a stream of oxygen-free gas (20% CO_2_, 80% N_2_, and 5% hydrogen). After the gassing procedure, the media were introduced into the anaerobic chamber. Cysteine HCl and NaHCO_3_ were added, and the culture medium was dispensed into anaerobic Balch tubes (10 mL of media per tube). The tubes were capped with rubber stoppers, tightly sealed with aluminum seals, autoclaved at 121 °C for 30 min, and finally, cooled to room temperature. After cooling, vitamin B12 and DiNP solutions were filtered-sterilized and injected into the Balch tubes containing *Bacteroides* minimal media.

### 2.8. Colonic Sample Collection and DNA Extraction for 16S rRNA Sequencing

Colonic samples (*n* = 5 per treatment group, 15 total) were collected within 10 min of euthanasia. Colonic samples (1 to 2 pellets/tube) were collected into Eppendorf tubes. All samples were immediately placed in liquid nitrogen and then stored in the laboratory at −80 °C prior to analysis.

Microbial DNA was extracted from colonic samples using the DNeasy PowerLyzer PowerSoil Kit by QIAGEN. The protocol uses a bead-beating method to extract the DNA. DNA extraction was acquired by following the manufacturer’s instructions. DNA concentration was measured using NanoDrop-1000 spectrophotometer. The full-length 16S region was amplified using a Fluidigm Biomark HD and sequenced on a Pacific Biosciences Sequel IIe at the Functional Genomics and DNA Services units at the Roy J. Carver Biotechnology Center at the University of Illinois.

### 2.9. 16S Microbiome Analysis

The High-Performance Computing in Biology (HPCBio) Group at the University of Illinois completed the 16S gene sequencing data analyses. The Fluidigm data targeted the full length of 16S (V1-V9) rRNA gene. The 16S rRNA genes were processed using the TADA Nextflow-based workflow (https://github.com/h3abionet/TADA, accessed date 25 February 2021), which implements the DADA2 workflow for denoising and dereplicating reads to generate amplicon sequence variants (ASVs) [29]. Primer sequences were removed, and reads were truncated to 240 nucleotides (nt) in length after minimal quality trimming. The remaining sequences with unclassified bases were removed. Default steps were used to denoise reads, merge paired reads into a single amplicon sequence, and dereplicate into ASVs. This was followed by taxonomic assignment using the Silva v132 database [30] and the DADA2 implementation of the RDP Classifier [31]. Using DECIPHER, multiple sequence alignment of the resulting ASV sequences was performed. To produce a maximum likelihood tree used in data analysis steps, a midpoint-rooted Fasttree [32] phylogenetic analysis was conducted. Using the package phyloseq v. 1.34.0, raw counts, taxonomic assignments, and the phylogenetic tree for the ASVs were imported into R v. 4.0.3 (R Core Team 2020) [33]. Mitochondrial, chloroplast, and unassigned phylum ASVs were filtered out, leaving 1703 ASVs. We then agglomerated ASVs that had extremely close phylogenetic distances <0.05, adding their counts together, leaving 505 “taxa”. Taxa were then discarded if they were detected in fewer than two samples, leaving 432 taxa. Alpha diversity was estimated separately using the sets of 1703, 505, and 432 taxa. The alpha diversity metrices included Observed species richness, Chao1, Shannon, Simpson, Inverse Simpson, and FaithPD estimates. All other analyses were performed on the set of 432 taxa, including beta diversity, PERMANOVA, and tests for differential abundance. The beta-diversity metrices included Bray–Curtis, UniFrac and Weighted UniFrac estimates. Differential abundance was estimated using DESeq2 v. 1.30.0 using the Wald test, with the “local” method for fitting dispersions and the “poscounts” method for estimating size factors. We further agglomerated the taxa to Phylum (six taxa), Family (25 taxa) and Genus (53 taxa) levels and tested whether the relative abundances of these groups differed between control and the two DiNP treatments, using the same approach with DESeq2 [34].

### 2.10. Identification of DiNP-Degrading Bacteria

The identification and classification of bacterial organisms at the species level were determined through Sanger sequencing of the 16S ribosomal RNA (rRNA) gene. To conduct Sanger sequencing of the 16S rRNA gene, microbial genomic DNA was isolated from the Balch tubes containing a single species of bacteria (or in other words were isolated from the colon and were able to grow on minimal culture medium supplemented with DiNP as the sole carbon source) using the QIAGEN DNeasy PowerLyzer PowerSoil Kit according to manufacturer’s instructions. The concentration of the extracted DNA was determined through Nanodrop. Following DNA isolation and concentration determination, polymerase chain reaction (PCR) was carried out using Phusion^TM^ High-Fidelity DNA Polymerase, deoxyribonucleotide triphosphate (dNTP) mix, 27F primer (5′-AGAGTTTGATCMTGGCTCAG-3′), 1492R primer (5′-GGTTACCTTGTTACGACTT-3′), and water. Initial denaturation (98 °C for 30 s, 1 cycle), denaturation (98 °C for 5–10 s, 25–35 cycles), annealing (58 °C for 10–30 s, 25–35 cycles), extension (72 °C for 15–30 s/kb, 25–35 cycles), and final extension (72 °C for 5–10 min, 1 cycle) was carried out according to the manufacturer’s protocol. At the completion of PCR, the amplified DNA was held at 4 °C until further processing. The quality of the DNA was confirmed as a single band was produced during agarose gel electrophoresis. The QIAquick^®^ PCR Purification Kit by QIAGEN was used to clean DNA by removing primers, nucleotides, salts, and enzymes from the DNA samples.

Sanger sequencing was performed at Roy J. Carver Biotechnology Center at the University of Illinois Urbana-Champaign. Sequencing reactions were set up as follows: 8 μL of water, 4 μL of 5M betaine, 2 μL of 5X Sequencing Buffer (Thermo Fisher, Waltham, MA, USA), 1.5 μL of BigDye^®^ Terminator v3.1 (Thermo Fisher), 0.5 μL dGTP BigDye^®^ Terminator v3.0 (Thermo Fisher), 2 μL of primer, and 2 μL of template per sample. Thermal cycling was performed at 98 °C for 5 min followed by 40 cycles of 98 °C for 15 s, 45 °C for 5 s and 60 °C for 4 min. Reaction products were cleaned using a DyeEx kit (Qiagen) and denatured at 95 °C for 2.5 min. Products were loaded onto an Applied Biosystems 3730xl DNA Analyzer equipped with a 50 cm 96-capillary array and running POP-7TM polymer (Thermo Fisher). Samples were run using a modified version of the default LongSeq50_POP7 run module, where injection time was increased to 25 s and run time was decreased to 5040 s. Samples were analyzed on the SeqA_7 software (Thermo Fisher).

Finally, sequences were entered into the nucleotide Basic Local Alignment Search Tool (BLAST) database available at the National Center for Biotechnology Information (NCBI). The sequences were searched and compared to the database using Megablast, which is optimized for similar sequences, to determine the species of the bacteria.

### 2.11. Statistical Analysis

Graphpad Prism 9.0.2 was used to determine outliers and perform statistical analyses. Outliers were identified and removed from analysis using the ROUT method. The data were expressed as means ± standard error of the means (SEM).

The Shapiro–Wilk test was used to analyze the data for normality and lognormality. Normally distributed data that met the assumption for homogeneity of variance (HOV) were analyzed using one-way analysis of variance (ANOVA) followed by a post hoc analysis—Dunnett’s two-sided test. The nonparametric Kruskal–Wallis test was used to analyze data that were not normally distributed and did not meet the assumption for HOV. The Dunn’s multiple comparisons test was conducted for post hoc analysis. 

## 3. Results

### 3.1. Alpha and Beta Diversity

Measurements of alpha and beta diversity are distinct high-level measurements to access a broad change in the composition of microorganisms. Specifically, alpha diversity measurements examine the richness or distribution within a single group, whereas beta diversity measurements examine the similarity or dissimilarity between treatment groups. Multiple metrics including Observed, Chao1, Shannon, Simpson, Inverse Simpson, and FaithPD were used to measure alpha diversity of the colonic microbiota communities of adult female mice exposed to vehicle control, 20 µg/kg/day of DiNP, or 200 µg/kg/day of DiNP. Different metrices were used because each metric reflects different aspects of community heterogeneity. DiNP treatment did not significantly alter alpha diversity compared to control (Supplementary Figure S1). Multiple metrics including UniFrac, weighted UniFrac, and Bray–Curtis were also used to measure beta diversity in the colonic microbiota of control and DiNP-exposed mice. DiNP exposure did not significantly impact beta-diversity compared to control (Supplementary Figure S2).

### 3.2. Taxonomic Results

**Figure 1:**
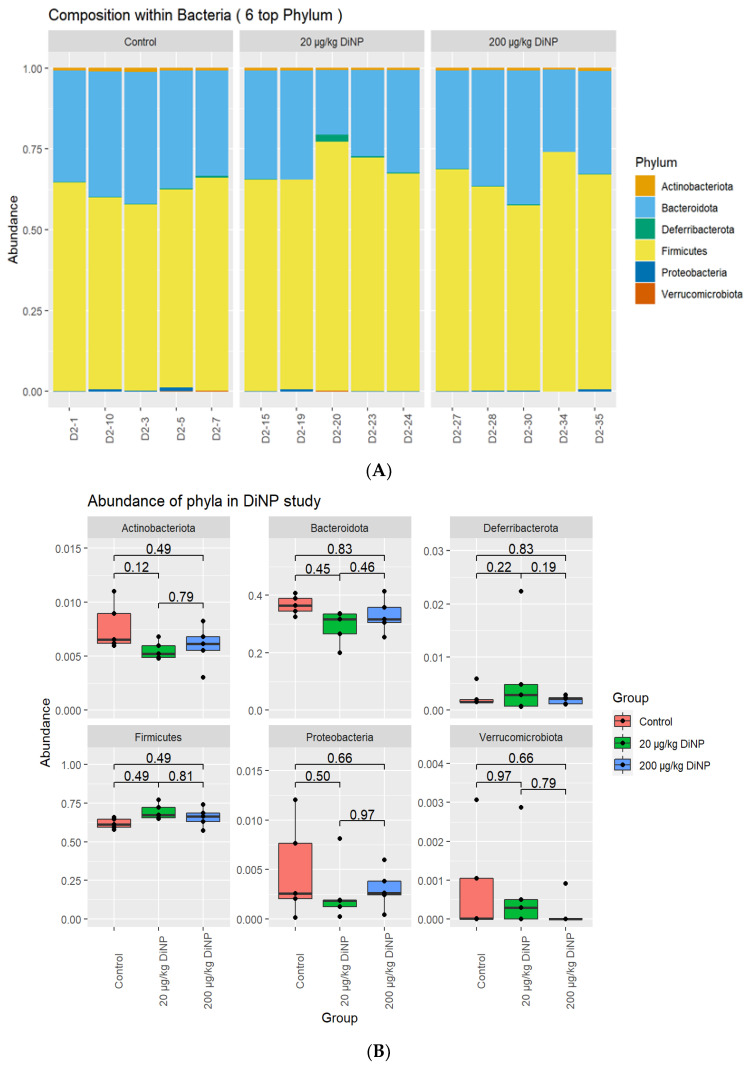
Phyla quantification. Overall relative abundance composition of colonic bacteria for phyla are identified in each sample (**A**), and the relative abundance of each phylum by treatment group is summarized in (**B**).

Six phyla were identified in the samples: Actinobacteriota, Bacteriodota, Deferribacterota, Firmicutes, Proteobacteria, and Verrucomicrobiota. Firmicutes and Bacteriodota composed >90% of the phyla composition. DiNP exposure at 20 and 200 did not significantly alter the relative abundance of Actinobacteriota, Bacteriodota, Deferribacterota, Firmicutes, Proteobacteria, and Verrucomicrobiota compared to control (Figure 1). As a side note, PERMANOVA *p*-values for association of treatment and library size with distance matrices were not statistically different between treatment groups and control (Table 3).

**Figure 2:**
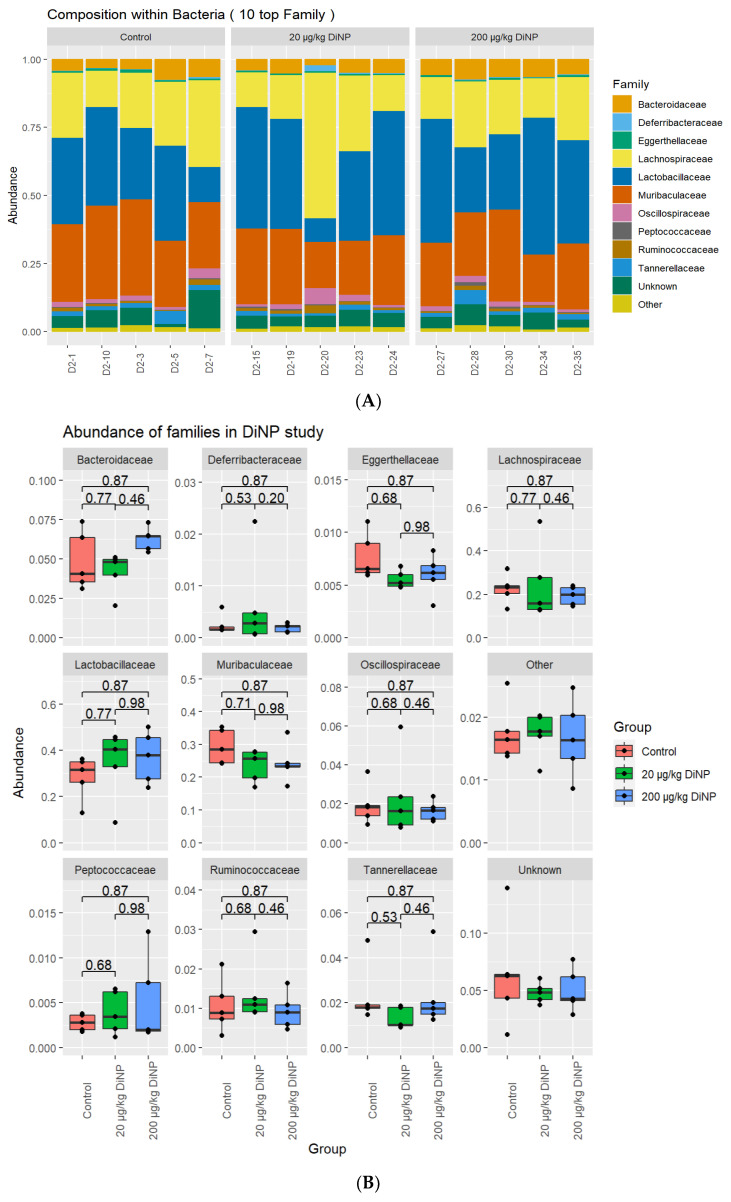
Overall family abundance composition of each sample in control and treatment groups (**A**) and relative abundance of each family by treatment group (**B**). *n* = 5/treatment group.

*Bacteriodaceae*, *Deferribacteraceae*, *Eggerthellaceae*, *Lachnospiraceae*, *Lactobacillaceae*, *Muribaculaceae*, *Oscillospiraceae*, *Peptococcaceae*, *Ruminococcaeae*, and *Tannerellaceae* were detected among the families. However, DiNP exposure did not significantly alter these families compared to the control (Figure 2).

**Figure 3:**
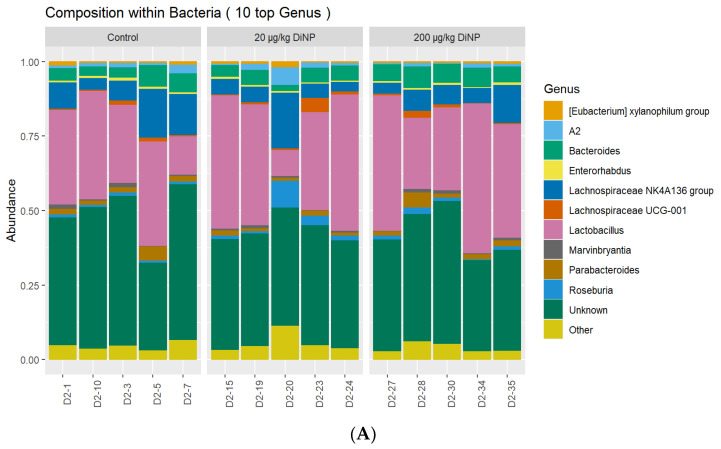

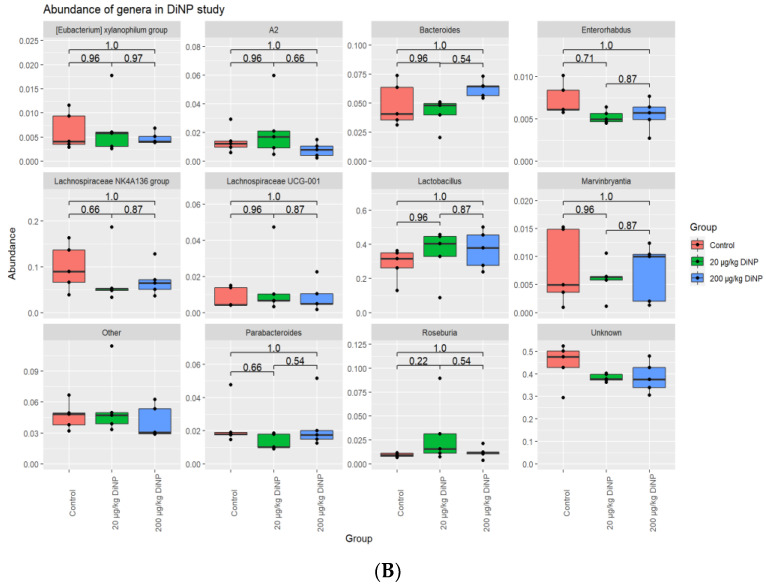
Overall genus abundance composition of each sample in control and treatment groups (**A**) and relative abundance of each family by treatment group (**B**). *n* = 5/treatment group.

**Figure 4:**
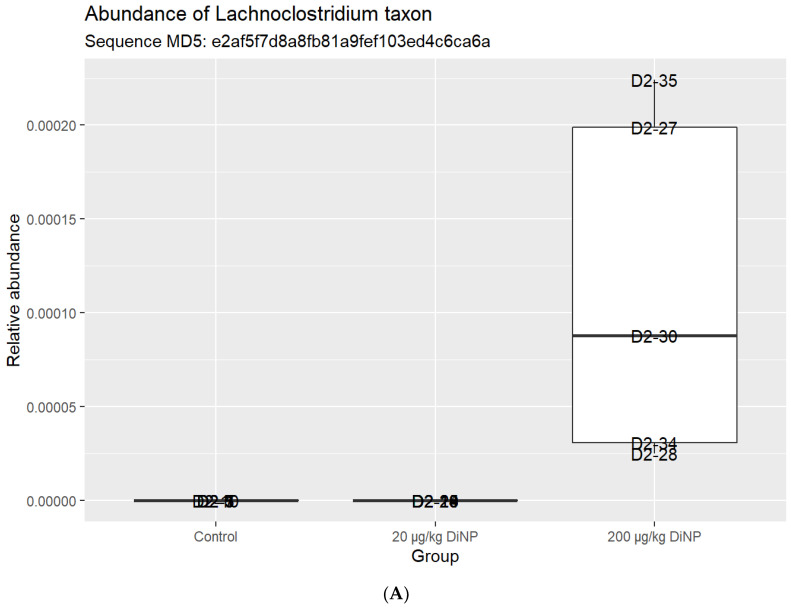

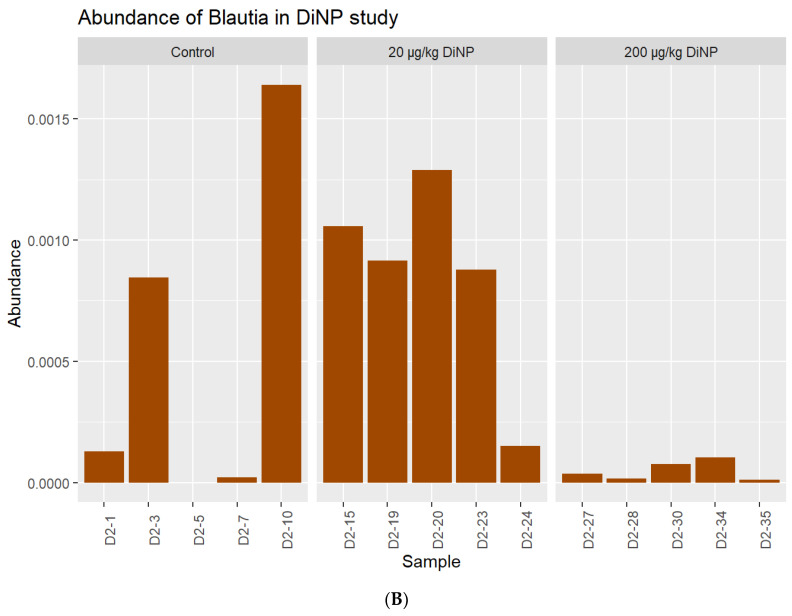
Abundance of *Lachnoclostridum* (**A**) and *Blautia* (**B**) in control and treatment groups.

Similarly, DiNP exposure did not significantly alter the following genera compared to control: *Eubacterium xylanophilum*, *A2*, *Bacteroides*, *Enterohabdus*, *Lachnospiraceae NKA136*, *Lachnospiraceae UCG*-*001*, *Lactobacillus*, *Marvinbryantia*, *Parabacteroides*, and *Roseburia* (Figure 3). Other genera were tested, but not listed in the composition plot (Table 4). Interestingly, among the ten agglomerated *Lachnoclostrium* taxa, we identified one that was present in the 200 µg/kg DiNP treatment group but was absent in the control and 20 µg/kg DiNP treatment groups (Figure 4). Finally, the relative abundance of *Blautia* was higher in the control and 20 µg/kg DiNP groups compared to the 200 µg/kg DiNP group (Figure 4). A summary of the number of taxa that were significantly altered by DiNP treatment compared to control is summarized in Table 5.

### 3.3. DiNP-Degrading Microbes Residing in the Colon

Genomic DNA was isolated from the bacteria that were able to utilize DiNP as the sole carbon source. The 16S rRNA gene was amplified by PCR using 27F and 1492R primers and the sequence was determined by Sanger sequencing. Therefore, Sanger sequencing revealed several bacteria from the colon that were capable of using DiNP as a carbon source. The following microbes were identified as DiNP-degraders: *Proteus mirabilis*, *Desulfitobacterium hafniense*, and *Paenibacillus barengoltzii*. Out of three microbes identified to degrade DiNP, Proteus mirabilis was the most abundant in degrading DiNP. See Table 6 for more strain details.

### 3.4. Immunohistochemistry of Cytokines and Other Factors Playing a Role in the Intestinal Immunology

**Figure 5:**
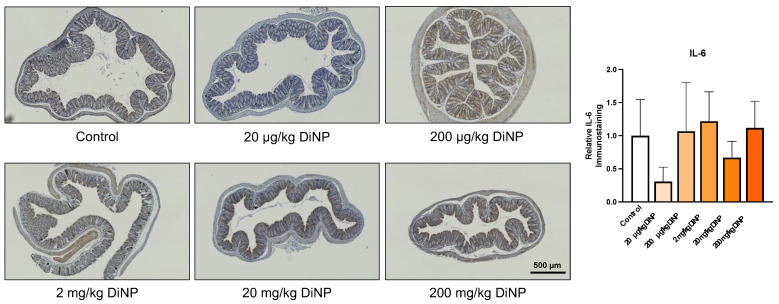
Interleukin-6 (IL-6) immunohistochemistry in the distal colon. Representative immunohistological images are displayed below at 5X objective. All DiNP treatment groups were compared to control. Quantification of IL-6 is in the graph on the right. The data are presented as means ± standard error of the mean (SEM). *n* = 4–6 samples/group.

**Figure 6:**
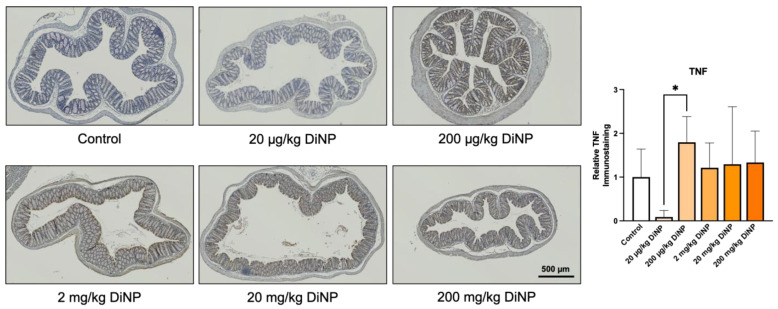
Tumor necrosis factor alpha immunohistochemistry in the distal colon. Representative immunohistological images are displayed below at 5X objective. All DiNP treatment groups were compared to control. Quantification of TNF is in the graph on the right. The data are presented as means ± standard error of the mean (SEM). *n* = 4–6 samples/group. The asterisk (*) indicates *p* < 0.05.

Two proinflammatory cytokines were examined in colons from DiNP treated and control mice via immunohistochemistry: tumor necrosis factor alpha (TNF) and interleukin-6 (IL-6). DiNP exposure did not significantly change levels of IL-6 and TNF compared to the control (Figure 5 and Figure 6). Interestingly, a significant difference was detected between the 20 µg/kg and 200 µg/kg DiNP treatment groups (*p* = 0.03). Adaptive immune cells were also examined in colons from DiNP treatment and control mice. We observed that CD3, a marker for T-lymphocytes, was intensely stained in a portion of the colonic patches and primarily negative or faintly positive outside of the colonic patches (Supplementary Figure S3). Therefore, we did not quantify CD3 immunostaining in the distal colon because it was largely negative outside the colonic lymph node patches.

**Figure 7:**
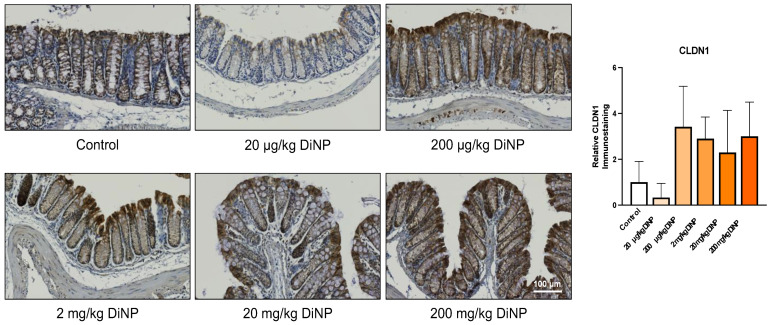
Claudin1 immunohistochemistry in the distal colon. Representative immunohistological images are displayed below at 20X objective. All DiNP treatment groups were compared to control. Quantification of CLDN1 is in the graph on the right. The data are presented as means ± standard error of the mean (SEM). *n* = 4–6 samples/group.

**Figure 8:**
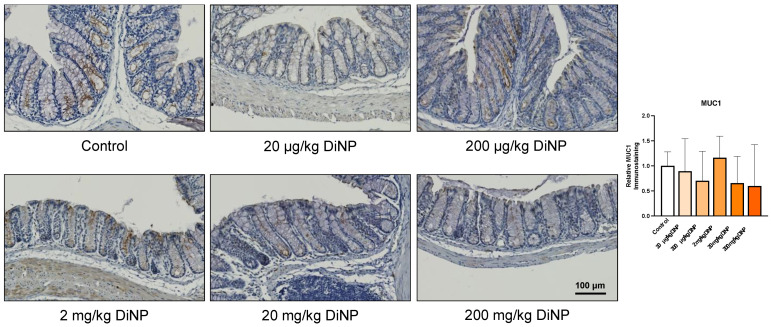
Mucin1 immunohistochemistry in the distal colon. Representative immunohistological images are displayed below at 20X objective. All DiNP treatment groups were compared to control. Quantification of MUC1 is in the graph on the right. The data are presented as means ± standard error of the mean (SEM). *n* = 4–6 samples/group.

Next, we examined factors that play a role in the intestinal immune microenvironment such as gut barriers (i.e., Claudin 1) and mucins (i.e., Mucin 1). Claudin 1 (CLDN1) supports intercellular barrier function of tight junctions, and mucin 1 (MUC1) is located on the cell surface to provide a barrier to infection. DiNP exposure did not significantly alter protein levels or CLDN1 and MUC1 compared to control (Figure 7 and Figure 8).

## 4. Discussion

Phthalate exposure has been shown to alter the gut microbiome in multiple studies. A review by Chiu et al. summarizes many of the phthalate exposure studies that alter the gut microbiome [3]. Briefly, DEHP, DEP, MEHP, DMP, and DBP exposure have been shown to alter the gut microbiota in a variety of models including rats [35], mice [7,8], and fish [5,6]. Human infant data have also shown that medically relevant doses of DEHP altered the gut microbiota in males and females [9]. Therefore, the finding that DiNP exposure alters the gut microbiota in female mice in the current study is consistent with previous studies on other phthalates.

Specifically, the current study revealed that DiNP exposure significantly altered the relative abundance of *Blautia* and one taxon within *Lachnoclostridum* compared to the control. As shown in Figure 5, *Blautia* has a very interesting trend such that the samples in the 20 µg/kg DiNP group have relatively high levels of *Blautia* and the samples in 200 µg/kg DiNP group have relatively low levels of *Blautia* compared to control. Interestingly, three of the five mice in the control group had relatively low levels of *Blautia*, and two of the five samples had relative high levels of *Blautia.* It is possible that the control group contains a heterogenous population of *Blautia* and that specific doses of DiNP promote or inhibit its growth. It is also worth noting that variations as observed in the control group are common in the gut microbiome literature [36].

*Lachnoclostridium*, a Gram-positive, obligately anaerobic spore-forming rod, ranks in the genus taxa and falls under the phyla Firmicutes. Interestingly, *m3* from a *Lachnoclostridium* species can be used as a diagnostic marker for colorectal adenoma and cancer [37]. The *Lachnoclostridium* genus includes microorganisms from several other clostridial clusters (e.g., *Clostridum* XIVa and *Clostridium* IV) and *Lachnospiraceae* [38,39]. *Clostridum* XIVa and *Clostridium* IV have been reported to regulate intestinal homeostasis, strengthen intestinal barriers, and alleviate dysfunctions and disorders in the intestine [40,41]. Although *Clostridium* is largely regarded for its positive health benefits, several species within *Clostridium* secrete toxins. For instance, *Clostridium perfringens* (*C. perfringens*) secrete alpha-toxins and enterotoxins and may cause necrotizing enteritis with necrotic jejunitis and segmental hemorrhages [42]. *Clostridum difficile* secrete toxins A and toxins B, which also leads to foodborne illnesses such as *C. perfringens* [43]. Overall, DiNP exposure significantly increased one taxon of *Lachnoclostridium* compared to control, but it is difficult to speculate about the role of *Lachnoclostridium* in the colon. This is because different species within the same genus are regarded as probiotics or pathogens, meaning they are either beneficial or destructive to the gut, respectively.

In addition to changes in the relative abundance of one agglomerated taxon within *Lachnoclostridium*, DiNP exposure significantly decreased *Blautia* compared to the control. *Blautia*, an anaerobic bacterium, has been reported to be significantly correlated with visceral fat accumulation in women and men [44], type 2 diabetes [45], and reduced morbidity from graft-versus-host disease [46]. Similar to *Lachnoclostridium*, some species of *Blautia* have probiotic properties. With at least 20 species with valid published names in this genus, it is also challenging to hypothesize about the role and function of *Blautia* in DiNP-exposed colons.

Although DiNP exposure significantly altered the relative abundance of *Blautia* compared to the control, the colonic microbiota on the whole did not significantly change as indicated by non-significant results in beta-diversity. A previous study reported that DiNP exposure significantly increased MUC2 (a marker for goblet cells) protein levels in the colon [23], which may possibly explain why there are no distinct microbial profiles between the control and treatment groups. It is possible that the increased differentiation of goblet cells increased mucus and mucin production, which could trap microorganisms. To determine whether this is possible, future studies would need to repeat the experimental design and conduct 16S rRNA gene sequencing on mucosal contents in the colon. Another possibility is that chronic exposure or higher doses of DiNP are needed to see significant changes in the colonic microbiome as a whole.

In the current study, DiNP exposure did not significantly alter protein levels of TNF, IL-6, MUC1, and CLDN1 compared to control. Although the select immune and immune-related biomarkers were not altered in response to DiNP, previous studies have shown that other cytokines and markers of tight junctions and mucins are significantly altered by DiNP treatment [3,23]. For instance, one study showed that subacute exposure to DiNP in female mice significantly increased expression of *Tnf* (a proinflammatory cytokine) and borderline increased *Ifng* (a proinflammatory cytokine) compared to control [3]. Furthermore, DiNP exposure significantly decreased protein levels of a cytokine called soluble intercellular adhesion molecular-1 (sICAM-1) compared to the control [3].

Protein levels of CLDN1 were not changed between DiNP treatment groups and control in the current study, and this corresponds to a previous study that showed that expression of *Cldn1* was not altered between DiNP treatment and the control [3]. Although the protein levels of CLDN1 were not altered in the current study, previous studies have shown that subacute exposure to DiNP in female mice significantly decreases the expression of *Zo-3*, which is another marker for tight junctions [3].

The current study also revealed that DiNP exposure did not impact protein levels of MUC1. Similarly, a previous study reported that DiNP exposure did not impact gene expression of *Muc1* compared to control [23]. However, DiNP exposure significantly increased a different marker for mucins, Mucin 2 (MUC2), compared to the control. MUC2 is the primary mucin and component of the protective mucus layer in the colon, which is why the MUC2 staining from a previous study [23] is stronger than the MUC1 staining from the current study.

Although moderate effects in the colonic microbiota were observed with DiNP exposure, the study had some limitations. First, we did not include metabolomic studies to examine whether DiNP effected microbiota-derived metabolites such as short-chain fatty acids, bile acids, amino acids, indole and tryptophan derivatives, and trimethylamine N-oxide. Another limitation of the current study is the lack of shotgun metagenomic sequencing to gain further understanding on the microbial community and insights on function. The third limitation of the current study is the lack of genome construction to determine whether the identified anaerobic bacteria contain enzymes that degrade DiNP. Finally, the collection of fecal samples before and throughout the course of the experiment would have been helpful to establish baseline differences in the gut microbiota. By tackling these limitations, these studies in addition to the current study could create the foundation for a therapeutic targeted toward treating DiNP-induced microbial changes in the colon.

## 5. Conclusions and Future Directions

In summary, this study shows that subacute exposure to DiNP in adult female mice alters the gut microbiota and that certain anaerobic gut bacteria can use DiNP as a carbon source. Further studies are needed to understand and elucidate the function of the DiNP-degrading bacteria identified in this current study and how they impact colon function and its immune microenvironment. Therefore, one of our future directions is to transplant DiNP-degrading bacterial species into germ-free mice and evaluate colon anatomy and physiology. Although the majority of the microbes in the colon prefer anaerobic environments, it is possible that aerobic DiNP-degrading bacteria degrade DiNP. Therefore, future studies should also explore whether aerobic DiNP-degrading bacteria impact colon health.

## Figures and Tables

**Table 1:** Components for liquid anaerobic diluent for storing microbes temporarily. Fresh colonic samples were stored in anaerobic Balch tubes containing anaerobic diluent for no more than two hours.

Anaerobic Diluent (Modified from C.S. McSweeney et al., 2005)	
8% Na_2_CO_3_ solution	5 mL
Solution No. 1 (See below)	3.8 mL
Solution No. 2 (See below)	3.8 mL
0.1% Resazurin	0.1 mL
ddH_2_O	87.3 mL
Total Volume	100 mL
Solution No. 1—g/L in dH_2_O	
KH_2_PO_4_	6 g
Solution No. 2—g/L in dH_2_O	
CaCl_2_–2H_2_O	1.6 g
KH_2_PO_4_	6 g
NaCl	12 g
(NH_4_)_2_SO_4_	6 g
MgSO_4_–7H_2_O	2.5 g

**Table 2:** Components for maintaining and cultivating colonic microbes. The table below lists the materials used to maintain and cultivate DiNP-degrading microbes obtained the mouse colon. The protocol was adapted from Bacic and Smith [28].

Bacteroides Defined Minimal Media (Modified from Bacic and Smith, 2013)	
Mineral 3B solution	50 mL/L
Cysteine hydrochloride	1 g/L
Hemin solution	10 mL/L
0.01% vitamin B12	1 mL/L
10% DiNP solution	10 mL/L
Iron (II) solute (FeSO4) solution	1.5 mL/L
7% NaHCO3	14.4 mL/L
0.1% resazurin	1 mL/L
Distilled water	Add to make 1 L
Mineral 3B solution—g/L in dH_2_O	
KH_2_PO_4_	18 g/L
NaCl	18 g/L
MgCl_2_•6H_2_O	0.4 g/L
CaCl_2_•2H_2_O	0.52 g/L
CoCl_2_•6H_2_O	0.02 g/L
MnCl_2_•4H_2_O	0.20 g/L
NH_4_Cl	10 g/L
Na_2_SO_4_	5 g/L

**Table 3:** PERMANOVA *p*-values for association of treatment and library size with distance matrices. In the first column labeled “study”, “DiNP” indicates all samples, “DiNP20” indicates controls and 20 µg/kg treatment only, and “DiNP200” indicates control and 200 µg/kg treatment only.

Study	Variable	Bray	UniFrac	Weighted UniFrac
DiNP	Group	0.787	0.599	0.588
DiNP	Library Size	0.328	0.569	0.712
DiNP20	Group	0.695	0.752	0.338
DiNP20	Library Size	0.340	0.753	0.605
DiNP200	Group	0.520	0.781	0.386
DiNP200	Library Size	0.612	0.206	0.727

**Table 4:** Genus detected in control and DiNP treatment groups. Cells and text highlighted in green indicate borderline significance (*p* ≤ 0.10).

Genus	Adjusted *p*-Value		
	Control vs. 20 µg/kg DiNP	Control vs. 200 µg/kg DiNP	20 µg/kg DiNP vs. 200 µg/kg DiNP
*GCA-900066575*	0.9579	0.9965	0.873
*Lachnospiraceae UCG-006*	0.9253	0.9965	0.5428
*Lachnoclostridium*	0.9279	0.9965	0.6572
*[Eubacterium] ventriosum group*	0.9579	0.9655	0.6572
*Lachnospiraceae FCS020 group*	0.9579	0.9965	0.873
*Roseburia*	0.2213	0 9965	0.5428
*Blautia*	0.9579	0.1565	0.0514
*Acetatifactor*	0.9579	0.9965	0.873
*Lachnospiraceae NK4A136 group*	0.6583	0.9965	0.873
*Marvinbrvantia*	0.9579	0.9965	0.873
*Lachnospiraceae UCG-001*	0.9579	0.9965	0.873
*A2*	0.9579	0.9965	0.6572
*[Eubacterium] xylanophilum group*	0.9579	0.9965	9713
*DefluviitaleaceaeUCG-011*	0.9579	0.9965	0.873
*Tyzzerella*	0.9579	0.9965	0.6572
*Tuzzerella*	0.9579	0.9983	0.873
*ASF356*	0.9579	0.9965	0.873
*Lachnospiraceae UCG-010*	0.9579	0.9965	0.9103
*Christensenella*	0.9579	0.9965	0.873
*Christensenellaceae R-7 group*	0 9579	0 9965	0.873
*Monoglobus*	0.6583	0.9965	0.5428
*Oscillibacter*	0.9579	0.9965	0.873
*Colidextribacter*	0.7136	0 9965	0.873
*Intestinimonas*	0.7084	0.9965	0.8842
*NK4A214 group*	0.9579	0.9965	0.873
*Papillibacter*	0.9579	0.9965	0.873
*UCG-005*	0.6583	0.9965	0.6522
*UCG-009*	0.9579	0.9965	0.873
*Butyricicoccus*	0.7136	0.9965	0.873
*Incertae Sedis*	0 9579	0.9965	0.6572
*Ruminococcus*	0.9579	0.3723	0.5428
*[Eubacterium] siraeum group*	0.9579	0 9965	0.9713
*Anaerotruncus*	0.6583	0.9965	0.6047
*Negativibacillus*	0.9579	0.9965	0.873
*Harryflintia*	0.9579	0.9965	0.873
*Paludicola*	9579	0.9965	0.98
*Candidatus Arthromitus*	0.9579	0.9965	0.873
*Anaerofustis*	0.9579	0.9965	0.9713
*Anaerovorax*	0.981	0.9965	0.9277
*[Eubacterium] nodatum group*	0.9579	0.9965	0.873
*Family XIlI AD3011 group*	0.9579	0.9965	0.873
*[Eubacterium] brachy group*	0.6583	0.9965	0.6572
*Family XIII UCG-001*	0.9579	0.9965	0 8730
*Lactobacillus*	0.9579	0.9965	0.873
*Erysipelatoclostridium*	0.7136	0.3782	0.873
*Enterorhabdus*	7136	0.9965	0.873
*Parvibacter*	0.7169	0.9965	0.5428
*Parasutterella*	0.7367	0.9965	0.873
*Mucispirillum*	0.7136	0 9965	0.6047
*Akkermansia*	0.9579	0.9965	0.873
*Alistipes*	0.9579	0.9965	0.873
*Parabacteroides*	0.6583	0.9965	0.5428
*Bacteroides*	0.9579	0.9965	0.5428

**Table 5:** Number of taxa with marginally significant (0.05 ≤ *p* < 0.1) abundances between each pair of treatments. “Up” means these are the number of taxa with marginally increased abundances between two treatments, “Down” indicates these are the number of taxa with marginally decreased abundances between two treatments, and “Non-significant” specifies the number of taxa that were not marginally significant.

	Control vs. 20 µg/kg DiNP	Control vs. 200 µg/kg DiNP	20 vs. 200 µg/kg DiNP
Up	0	1	3
Non-significant	430	429	425
Down	2	2	4

**Table 6:** The table lists anaerobic bacteria isolated from the colon and grown on agar plates with DiNP as the sole carbon source. Coverage indicates the number of nucleotide bases that align or cover the known reference base, and the percent identity for the forward and reverse primers (27F and 1492R) describes how similar the query sequence is to the target sequence.

DiNP-Degrading Bacteria Isolated from the Colon			
Species	Coverage (%)	Identity (%), 27F Primer	Identity (%), 1492R Primer
*Proteus mirabilis strain ATCC 29,906*	96	95.32	96.80
*Desulfitobacterium hafniense DCB-2*	89	92.41	99.07
*Paenibacillus barengoltzii strain NBRC 101,215*	98	95.97	97.94

## Data Availability

Not applicable.

## Supplementary Materials

The following are available online at https://www.mdpi.com/article/10.3390/toxics10020075/s1, Figure S1: Alpha diversity measures (A) and rarefaction (B); Figure S2: Beta-diversity of mice treated with control, 20 µg/kg DiNP, or 200 µg/kg DiNP; Figure S3: CD3 immunostaining in the colonic patches.
