# Supplementary material for: Isolation of DiNP-Degrading Microbes from the Mouse Colon and the Influence DiNP Exposure Has on the Microbiota, Intestinal Integrity, and Immune Status of the Colon
Source: Toxics. 2022 Feb 6;10(2):75. doi: 10.3390/toxics10020075 (PMC8877566; doi:10.3390/toxics10020075)
Supplement: Supplementary file 1 [file toxics-10-00075-s001.zip › toxics-1566328-supplementary.pdf]

toxics

MDPI

Supplementary Materials: Isolation of DiNP-Degrading Microbes from the Mouse Colon and the Influence DiNP Exposure Has on the Microbiota, Intestinal Integrity, and Immune Status of the Colon

Karen K. Chiu, Shah Tauseef Bashir, Ahmed M. Abdel-Hamid, Lindsay V. Clark, Mary J. Laws, Isaac Cann, Romana A. Nowak and Jodi A. Flaws

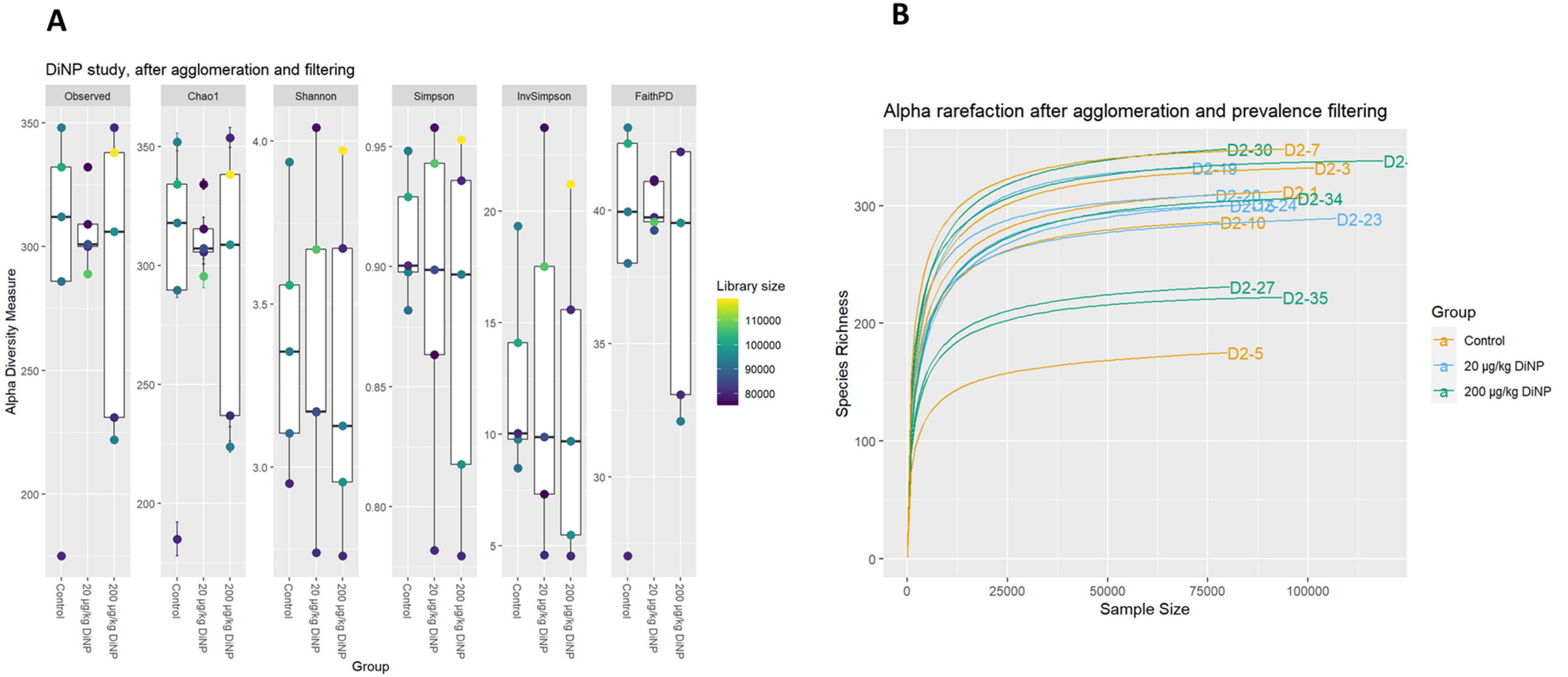

Figure S1. Alpha diversity measures (**A**) and rarefaction (**B**).

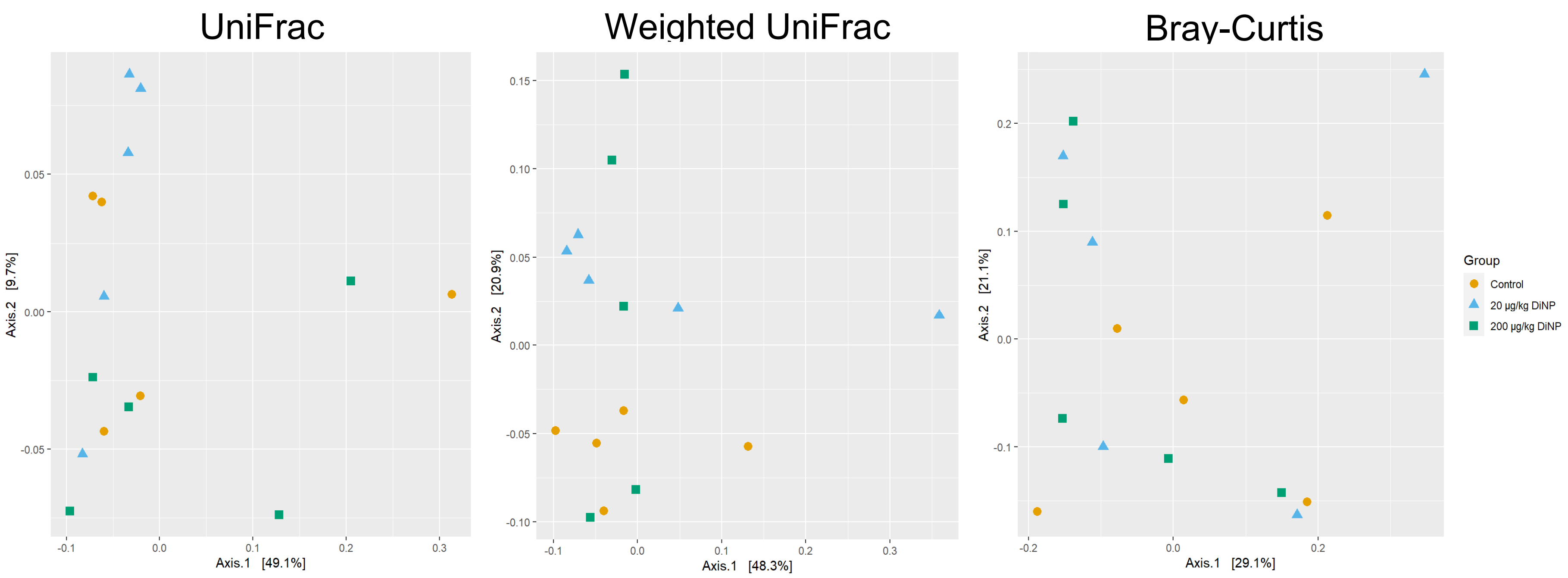

Figure S2. Beta-diversity of mice treated with control, 20 μg/kg DiNP, or 200 μg/kg DiNP. PCoA plots of three beta-diversity metrices (unweighted UniFrac, weighted UniFrac, and Bray Curtis) did not show significant separation between control and various DiNP treatment groups ($p > 0.05$). Orange circles represent control, blue triangles represent 20 μg/kg DiNP, and green squares represent 200 μg/kg DiNP (n = 5/treatment group).

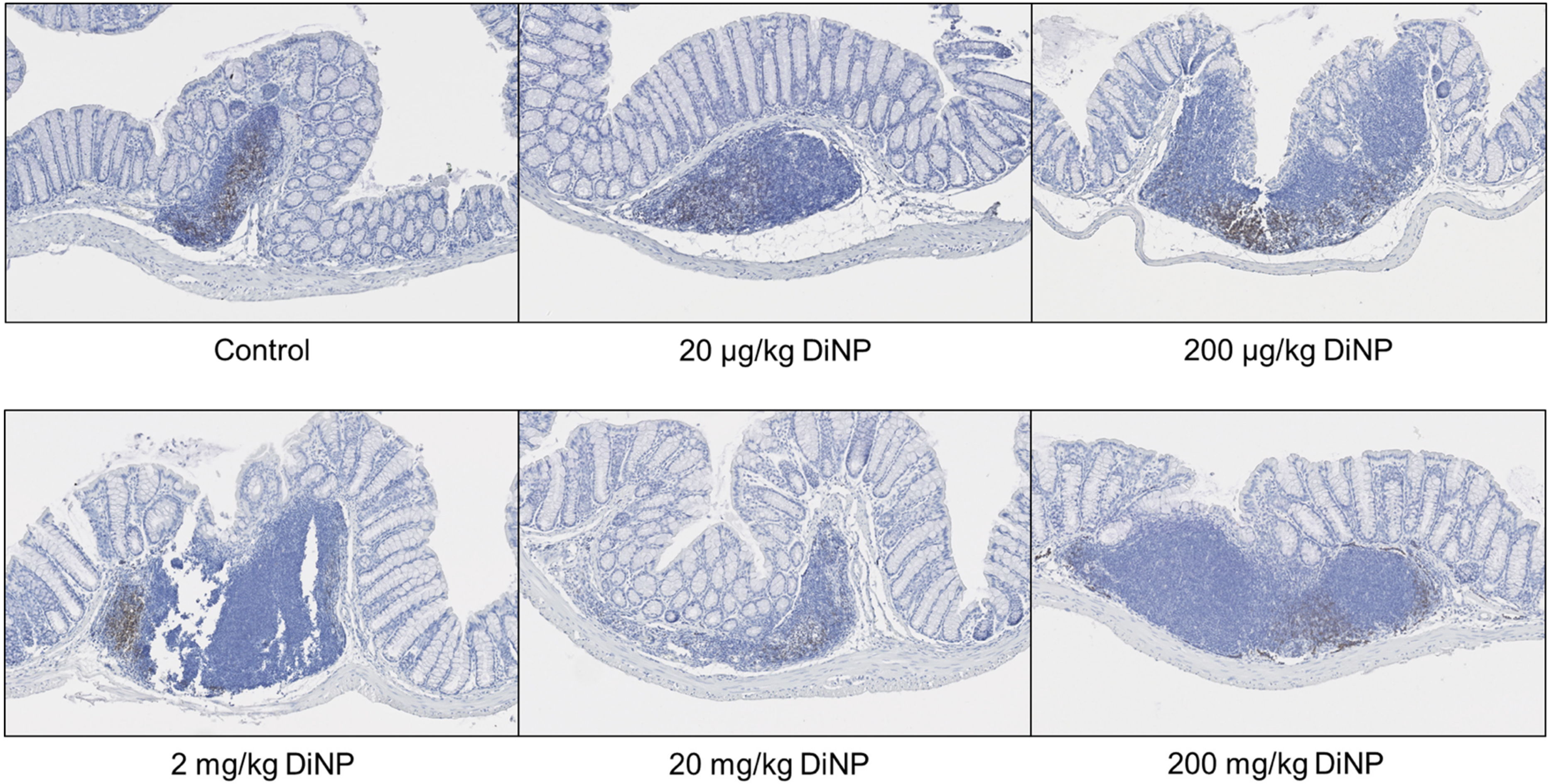

Figure S3. CD3 immunostaining in the colonic patches. Representative immunohistological images are displayed below. Positive immunostaining for CD3 was primarily shown in the colonic patches. Otherwise, CD3 immunostaining was primarily negative or faintly positive outside of the colonic patches. Statistical analysis was not completed for CD3 immunostaining because each group only had 1–2 colonic patches.
